# High-dose phytosterols supplementation improves lactation performance, modulates rumen microbiota, and reduces methane emission intensity in mid-lactation Holstein dairy cows

**DOI:** 10.14202/vetworld.2026.2339-2357

**Published:** 2026-06-05

**Authors:** Dong Li, Yinyin Kyawt, Qi Wang, Jian Gao, Rong Wang, Min Wang, Chen Duan, Donghai Lv, Weiyun Zhu, Metha Wanapat, Yanfen Cheng

**Affiliations:** 1 Laboratory of Gastrointestinal Microbiology, National Centre for International Research on Animal Gut Nutrition, Nanjing Agricultural University, Nanjing 210095, China; 2 Department of Animal Nutrition, University of Veterinary Science, Nay Pyi Taw 15013, Myanmar; 3 Key Laboratory of Forage Breeding-by-Design and Utilization, Institute of Subtropical Agriculture, Chinese Academy of Sciences, Changsha, Hunan 410125, People’s Republic of China; 4 Tropical Feed Resources Research and Development Center (TROFREC), Faculty of Agriculture, Khon Kaen University, Thailand 40002

**Keywords:** digestibility, lactation performance, methane mitigation, Phytosterols, rumen fermentation, volatile fatty acids

## Abstract

**Background and Aim:**

Enteric methane emission from dairy cows contributes substantially to greenhouse gas production and represents an inefficient loss of dietary energy. Phytosterols are plant-derived bioactive compounds with lipid-modulating and rumen fermentation-regulating properties; however, their effects on methane emission intensity and rumen microbial ecology in lactating dairy cows remain insufficiently explored. This study evaluated the effects of dietary phytosterols supplementation on lactation performance, nutrient digestibility, serum biochemical parameters, rumen fermentation characteristics, methane emission intensity, and rumen microbial composition in mid-lactation Holstein dairy cows.

**Materials and Methods:**

Thirty-four multiparous Holstein dairy cows with similar days in milk and milk yield were randomly assigned to either a control (CON) group or a phytosterols (PHY) group receiving 15 g/d of a commercial phytosterols product containing 5% active phytosterols. The experimental period lasted 50 days, including 7 days of adaptation and 43 days of data collection. Feed intake and milk yield were recorded daily. Milk composition, apparent nutrient digestibility, serum biochemical indices, rumen fermentation parameters, methane emission intensity, quantitative polymerase chain reaction, and 16S rRNA gene sequencing were analyzed. Methane and carbon dioxide emissions were measured using an automated head-chamber system.

**Results:**

Dietary phytosterols supplementation significantly improved milk yield, milk fat percentage, milk protein percentage, energy-corrected milk, and 3.5% fat-corrected milk compared with the CON group (p < 0.05). Apparent digestibility of organic matter, crude protein, neutral detergent fiber, and ether extract was also significantly enhanced. Serum glucose and blood urea nitrogen concentrations increased, whereas total cholesterol and low-density lipoprotein cholesterol concentrations decreased in the PHY group. Phytosterols supplementation significantly reduced methane emission intensity per kilogram of energy-corrected milk. Ruminal acetate proportion and acetate-to-propionate ratio decreased, whereas microbial crude protein and branched-chain volatile fatty acids increased. In addition, phytosterols altered rumen microbial composition by increasing the abundance of beneficial bacterial genera, including *Succinivibrionaceae* UCG-001 and *Prevotella*, while reducing methanogenic archaea, particularly *Methanobacteriota* and *Methanimicrococcus*.

**Conclusion:**

High-dose phytosterols supplementation improved lactation performance, enhanced nutrient utilization, modulated rumen microbial communities, and reduced methane emission intensity in mid-lactation dairy cows. These findings indicate that phytosterols may serve as a promising natural feed additive for improving dairy production efficiency while supporting methane mitigation strategies in sustainable dairy farming.

## INTRODUCTION

Methane (CH_4_), a potent greenhouse gas, has a global warming potential approximately 28 times greater than that of carbon dioxide (CO_2_) [1]. Ruminants represent the primary source of CH_4_ emissions in livestock production [2]. In particular, methane emitted from the gastrointestinal tract of dairy cows accounts for approximately 31.6% of total global agricultural methane emissions [3]. In the anaerobic rumen environment, a diverse microbial consortium ferments carbohydrates into volatile fatty acids (VFAs) and hydrogen (H_2_), whereas methanogenic archaea utilize H_2_ to reduce CO_2_ or other one-carbon substrates into CH_4_ [4]. This interspecies H_2_ transfer is thermodynamically essential because it prevents H_2_ accumulation and supports efficient fiber digestion; however, it also represents a substantial loss of dietary energy for the host animal [3]. During ruminal fermentation, approximately 2%–12% of gross dietary energy may be lost as CH_4_ [5]. Therefore, enteric methane production not only contributes significantly to global climate change but also decreases feed energy utilization efficiency, making methane mitigation a major priority in ruminant nutrition research.

To date, nutritional and management interventions have achieved only moderate reductions in enteric CH_4_ emissions, generally ranging from 2% to 15% [6]. Most currently available feed additives provide less than 20% methane mitigation, with the exception of certain chemical inhibitors such as 3-nitrooxypropanol, which can reduce methane emissions by approximately 20%–40% [5]. In contrast, phytogenic feed additives have gained increasing attention as sustainable alternatives for methane mitigation. Several plant-derived compounds, including tannins, saponins, and essential oils, have demonstrated the ability to reduce enteric CH_4_ production by redirecting fermentation hydrogen toward propionate synthesis and altering methanogen and protozoal populations within the rumen ecosystem [7-9]. Jadhav *et al*. [10] reported that supplementation with 0.8% tea saponins derived from *Camellia sinensis* during *in vitro* rumen fermentation reduced methane production by 29%–36%. These findings highlight the considerable potential of phytogenic bioactive compounds as environmentally sustainable methane mitigation strategies in dairy production systems.

Among these phytogenic compounds, phytosterols are naturally occurring bioactive steroid molecules found in plant cell membranes. Major phytosterol components include β-sitosterol, campesterol, and brassicasterol, which are abundant in oilseeds and forage crops [11]. Structurally similar to cholesterol, phytosterols competitively inhibit intestinal cholesterol absorption through interference with mixed micelle formation [12]. In both humans and animals, phytosterols are recognized for their cholesterol-lowering, anti-inflammatory, and lipid-modulating properties [13]. Although phytosterols are poorly absorbed in the gastrointestinal tract, they may exert substantial local effects on gut microbial activity and host metabolism [14]. Previous studies in ruminants have suggested that phytosterols may positively influence rumen fermentation characteristics and nutrient utilization. Xi *et al*. [15] demonstrated during *in vitro* fermentation that phytosterols increased dry matter digestibility and microbial protein synthesis while reducing ammoniacal nitrogen and lactate concentrations. Similarly, Lv *et al*. [16] reported through multi-omics analysis that phytosterols supplementation in periparturient dairy cows increased total ruminal bacterial abundance, enhanced microbial protein production, enriched fibrolytic bacteria such as *Fibrobacter succinogenes*, and increased propionate production. Furthermore, Zhao *et al*. [17] observed that phytosterols supplementation improved nutrient digestibility and altered ruminal bacterial abundance in Tibetan sheep. Collectively, these studies indicate that phytosterols may regulate rumen fermentation patterns, improve microbial efficiency, and potentially redirect metabolic hydrogen away from methanogenesis.

Despite the growing interest in phytosterols as functional phytogenic feed additives, substantial knowledge gaps remain regarding their application in lactating dairy cows under practical production conditions. Most previous investigations have primarily focused on *in vitro* rumen fermentation systems or physiological transition periods such as the periparturient stage, where metabolic demands and microbial dynamics differ considerably from those observed during mid-lactation. Consequently, the biological responses of established lactating dairy cows to high-dose phytosterols supplementation remain insufficiently characterized.

In addition, earlier studies mainly evaluated limited parameters such as nutrient digestibility, serum metabolites, or general microbial abundance, whereas integrated evaluation of methane emission intensity, ruminal fermentation characteristics, lactation performance, and ruminal microbial ecology has rarely been performed simultaneously within a single *in vivo* experiment. Information regarding the effects of phytosterols on methanogenic archaea, hydrogen-utilizing microbial pathways, and fermentation-driven methane mitigation mechanisms in dairy cows is particularly limited. Moreover, available studies have largely used relatively low phytosterol doses and did not assess the efficacy of commercially applicable supplementation levels under farm-like conditions.

Another important limitation in previous research is the lack of comprehensive microbial profiling using both quantitative polymerase chain reaction and *16S rRNA* gene sequencing to evaluate bacterial and archaeal community restructuring associated with methane reduction. The interaction between phytosterols supplemen-tation, rumen microbial shifts, VFA production patterns, and enteric methane emission intensity therefore remains poorly understood. Addressing these limitations is essential to determine whether phytosterols can serve as a practical nutritional strategy for simultaneously improving dairy production efficiency and reducing the environmental impact of dairy farming.

Therefore, the present study aimed to evaluate the effects of dietary supplementation with a β-sitosterol-rich commercial phytosterols product at 15 g/d on lactation performance, nutrient digestibility, serum biochemical parameters, rumen fermentation characteristics, methane emission intensity, and ruminal microbial composition in mid-lactation Holstein dairy cows. In addition, this study aimed to characterize the shifts in ruminal bacterial and archaeal communities using quantitative polymerase chain reaction and *16S rRNA* gene sequencing analyses. We hypothesized that high-dose phytosterols supplementation would improve nutrient utilization and lactation performance, shift rumen fermentation toward propionate production, modulate ruminal microbial ecology, suppress methanogenic archaea, and consequently reduce enteric methane emission intensity in dairy cows.

## MATERIALS AND METHODS

### Ethical approval

All study procedures were reviewed and approved by the Animal Care and Use Committee of Nanjing Agricultural University, Jiangsu, China (Approval No. SYXK 2024-0196). To minimize pain and stress to the cows during the experiment, all sampling procedures were performed by professional personnel with more than 15 years of on-farm experience, following standardized non-invasive or minimally invasive operating procedures. Ruminal intubation was performed gently throughout the process to avoid damage to the esophagus and rumen wall of the cows. During the experiment, the cows were housed in a temperature-controlled barn with comfortable stalls, manure was cleaned twice daily, sufficient clean drinking water was guaranteed, and continuous health monitoring was performed. No illness or culling occurred during the entire experimental period, and animal welfare was fully maintained throughout the study.

### Study period and location

This experiment was conducted from May 2024 to August 2024 at Changzhou Mingyuan Animal Husbandry Co., Ltd., China. The total experimental period lasted 50 days, including a 7-day adaptation period and a 43-day formal experimental period. Data regarding feed intake and milk yield were collected daily throughout the study period. Feed, fecal, and milk samples were obtained on days 30–35 for milk composition analysis and determination of apparent digestibility. Blood samples were collected on day 36 for serum biochemical analysis. Methane and carbon dioxide measurements were conducted until day 43. On day 37, ruminal fluid was collected through the oral cavity for evaluation of ruminal fermentation parameters, qPCR amplification, and *16S rRNA* gene sequencing analyses.

### Study design and dietary treatments

Thirty-four multiparous Holstein dairy cows with similar days in milk (207 ± 23 d), milk yield (35.4 ± 2.7 kg/d), initial body weight (676 ± 35 kg), and body condition score (3.35 ± 0.24) were selected for this study. The cows were stratified according to days in milk, milk yield, and parity and randomly assigned into two groups using a random number table, with 17 cows per group, receiving either 0 g (control [CON]) or 15 g/d of commercial phytosterols [16].

The phytosterols product used in this study was Noricon (Nanjing Nature Bio-Tech Co., Ltd., Jiangsu, China). The product contained 95% attapulgite carrier and 5% active phytosterols. The active phytosterol fraction consisted of β-sitosterol ≥ 44.71%, campesterol ≥ 27.23%, brassicasterol ≥ 16.63%, and total phytosterols ≥ 5%. All cows had unrestricted access to drinking water and total mixed ration (TMR) formulated according to NRC recommendations [18]. The composition of the TMR is presented in Table 1.

**Table 1:** Composition and nutrient levels of the basal diet (air-dry basis).

Item	Content (%)
Ingredient	
Barley ensilage¹	51.61
Beer vinasse	15.48
Alfalfa haylage²	9.68
Soybean and rapeseed meal	8.39
Concentrate supplement D-50³	8.39
Steam-flaked corn	5.45
Sodium bicarbonate (rumen buffer)	1.00
Nutrient level	
DM	45.74
CP	16.66
NDF	44.03
ADF	24.86
EE	2.60
Ca	0.89
P	0.51

1. Barley ensilage contained 34.8% DM and, on a DM basis, 10.1% CP and 49.7% NDF.

2. Alfalfa haylage contained 35.2% DM and, on a DM basis, 20.5% CP and 35.6% NDF.

3. Concentrate supplement D-50 pellets contained (% of grain mix, 100% basis): corn (31.00%), soybean hulls (6.00%), barley (10.00%), distillers dried grains with soluble (14.00%), double-low rapeseed meal (30.0%), laminated adsorbate (1.00%), calcium phosphate (1.00%), sodium bicarbonate (2.00%), limestone (5.00%), magnesium oxide (0.50%), and salt (2.50%). Per kilogram of premix, vitamins and minerals included: vitamin A, 65,000 IU; vitamin D, 6,000 IU; vitamin E, 1,100 IU; Fe, 1,800 mg; Cu, 95 mg; Zn, 480 mg; Mn, 170 mg; Se, 3.6 mg; I, 7.2 mg; and Co, 1.3 mg.

4. DM = Dry matter; OM = Organic matter; CP = Crude protein; NDF = Neutral detergent fiber; ADF = Acid detergent fiber; EE = Ether extract.

Data regarding feed intake and milk yield were collected daily. Feed, fecal, and milk samples were obtained on days 30–35 for milk composition analysis and determination of apparent digestibility. Blood samples were collected on day 36 for serum biochemical analysis. Seven dairy cows per group were randomly selected for methane and carbon dioxide measurement, which was conducted until day 43. On day 37, ruminal fluid was collected through the oral cavity to evaluate ruminal fermentation parameters, qPCR amplification, and *16S rRNA* gene sequencing. This multifaceted approach, including gas emissions measurement, microbial taxonomic analysis, fermentation characteristics detection, and production performance determination, provided a comprehensive evaluation rarely achieved in phytosterol ruminant research.

### Feed and milk samples

TMR samples were collected once weekly, thoroughly mixed, and stored at −80°C for subsequent nutrient composition analysis. Moisture, crude protein (CP), ether extract (EE), and ash contents were assayed according to the standard methods of Official Analytical Chemists (AOAC) [19]. Neutral detergent fiber (NDF) and acid detergent fiber were determined according to the protocols outlined by Van Soest *et al*. [20].

From morning feeding on day 30 to evening feeding on day 35, fresh fecal samples were obtained from each experimental cow and weighed at 4-h intervals (08:30, 12:30, 16:30, and 20:30), with four daily samplings covering the full 24-h period. The weight of each fecal sample per cow was recorded, and 100 g of the pooled and homogenized daily sample was transferred into petri dishes containing 10% nitrogen fixation sulfate, oven-dried at 65°C, and retained for subsequent nutrient analyses. The same analytical methods used for feed samples were applied for nutrient composition analysis of fecal samples.

The apparent digestibility of dietary nutrients was calculated using the endogenous indicator method with acid-insoluble ash as the endogenous marker according to the protocol outlined by Van Keulen and Young [21]. The specific calculation method was as follows:

Nutrient apparent digestibility = [1 − (dietary acid-insoluble ash content × fecal nutrient content)/(fecal acid-insoluble ash content × dietary nutrient content)] × 100%.

The experimental dairy cows were mechanically milked three times daily at 0630 h, 1430 h, and 2230 h, and daily milk yield was recorded. Starting on day 30 of the experiment, sampling of early, middle, and late milk fractions from each cow was initiated and continued for five consecutive days. Mixed milk samples were prepared according to the milk yield ratio (morning:noon:evening = 4:3:3). These mixed samples were placed into 50 mL centrifuge tubes containing potassium dichromate and stored at 4°C for subsequent determination of milk fat percentage, milk protein percentage, lactose, total solids, and non-fat solids using the FOSS-4000 analyzer (Foss, Hillerød, Denmark). The average milk composition values obtained over the 5-day sampling period for each cow were used for statistical analysis.

The formulas used for calculating 3.5% fat-corrected milk (FCM) and energy-corrected milk (ECM) were as follows:

3.5% FCM = 0.432 × milk yield + 16.216 × fat yield [22].

ECM = 12.96 × fat yield + 7.04 × protein yield + 0.3246 × milk yield [23].

### Blood collection and analyses

On day 36 of the trial, before morning feeding, 10 mL of blood was collected from the caudal vein of each experimental cow. The samples were left undisturbed at 4°C for 15 min and then centrifuged at 3,000 rpm (approximately 1,000 × *g*) for 10 min. The upper serum layer was collected, aliquoted into individual 2 mL centrifuge tubes, and cryopreserved at −80°C for subsequent testing.

Serum biomarkers analyzed included total protein, albumin, glucose, triglycerides, total cholesterol, low-density lipoprotein cholesterol, high-density lipoprotein cholesterol, blood urea nitrogen, glutamic oxalacetic transaminase, total antioxidant capacity, glutathione peroxidase, superoxide dismutase, and malondialdehyde. All indices were measured using commercial kits provided by Nanjing Jiancheng Bioengineering Institute, Nanjing, China.

### Rumen fluid collection and analyses

On day 37 of the experiment, before morning feeding, a large rumen tube was inserted orally and extended into the rumen. Initially, 30 mL of rumen fluid was extracted and discarded to avoid contamination from oral fluids or other external factors. Subsequently, 50 mL of rumen fluid was collected, and half of the sample was filtered through four layers of gauze and transferred into a beaker.

The pH of rumen fluid was measured using a portable pH meter calibrated with standard buffers (pH 4.0, 7.4, and 9.0), and the mean of three replicate readings was used for analysis. The lactic acid concentration was determined using a commercial lactic acid assay kit (Nanjing, China) through chemical colorimetry. Ammonia nitrogen concentration was determined using the phenol-sodium hypochlorite colorimetric method. Microbial CP concentration was determined using the Coomassie Brilliant Blue staining method [24].

The concentrations of VFAs, including acetate, propionate, butyrate, isobutyrate, valerate, and isovalerate, were determined using the internal standard method with an Agilent 7890B gas chromatograph (Agilent Technologies, Santa Clara, CA, USA). Thawed samples (1 mL) were mixed with 200 μL of 25% metaphosphoric acid solution, vortexed, and stored at −20°C for 24 h to precipitate proteins and other interfering substances. Before analysis, samples were thawed under running water, centrifuged at 4°C and 12000 rpm for 10 min, and the supernatant was filtered through a 0.22 μm syringe filter into a vial for gas chromatographic injection. Qualitative analysis was performed according to the retention times of characteristic peaks, and quantitative analysis was conducted using the internal standard method to obtain the absolute concentration and molar proportion of each VFA component.

### Enteric gas emissions

Enteric gas emissions (CH_4_ and CO_2_) were measured using an automated head-chamber (AHC) system developed by the Institute of Subtropical Agriculture, Chinese Academy of Sciences. The operating principle of this system was consistent with that of the GreenFeed system [25]. Based on the methodology described by Wang *et al*. [26], the AHC monitoring device was employed for data collection.

One week before formal measurements, the selected seven cows per group were trained to adapt to the system. Cows were guided to enter the head chamber and consume bait feed three times daily for 5–10 min each session until all cows could voluntarily enter the chamber without stress and complete feeding, thereby ensuring that no stress response interfered with measurement accuracy.

Seven cows from each group were randomly selected for testing, and each cow underwent two measurement rounds with eight time points recorded per round. The measurement schedule was as follows: 06:00 and 18:00 on day 1; 03:00 and 15:00 on day 2; 00:00 and 12:00 on day 3; and 09:00 and 21:00 on day 4. This time-point sequence was repeated from day 5 to day 8. This eight-time-point sampling strategy has been experimentally validated to represent more than 98% of the daily enteric methane emission pattern in dairy cows, thereby ensuring the reliability and representativeness of the gas measurement data obtained in this trial [25].

During AHC operation, granular bait feed consisting of corn (60%), carex (11.5%), alfalfa (20%), sugar (7%), salt (0.5%), and soybean oil (1%) was used to attract cows into the chamber and ensure proper head positioning during measurement. The bait feed was delivered from the feeding chamber at approximately 100 g/min and was used solely to attract cows into the chamber. Because the bait feed had no significant effect on nutrient intake or total feed intake, it was not included in feed intake calculations.

Methane concentration values were averaged over a 5-min measurement period for each cow, followed by a 2-min environmental gas measurement period. The AHC system was calibrated each morning using methane standard gas to ensure measurement accuracy. For comparative analysis, methane yield was normalized relative to average milk yield, 3.5% FCM, and ECM yields.

Only valid measurements in which the cow’s head remained completely inside the head chamber for more than 80% of the measurement period were retained. Ambient gas background concentrations measured simultaneously were subtracted from the sample gas concentration values. Outliers beyond ± 2 standard deviations from the mean concurrent measurements of the same cow were excluded. The coefficient of variation of repeated methane measurements in this experiment was 5.2%, indicating good repeatability of the measurements.

### Ruminal DNA isolation and qPCR amplification

Total DNA from ruminal microorganisms was isolated according to the protocol proposed by Xu *et al*. [27]. After DNA extraction, the quality of extracted DNA was evaluated using an ultra-microspectrophotometer, and OD260/280 values between 1.8 and 2.0 were considered acceptable. Qualified DNA samples were stored at −20°C until further analysis.

The qPCR reaction system was prepared using ChamQ Universal SYBR qPCR Premix (Vazyme Biotech Co., Ltd., Nanjing, China) and consisted of 10 μL Master Mix, 0.4 μL forward primer, 0.4 μL reverse primer, 7.2 μL double-distilled water, and 2 μL DNA template. The specific primer pairs targeting the *16S rRNA* genes of bacteria and archaea are listed in Supplementary Table 1.

The recombinant plasmid standard containing the target *16S rRNA* gene fragment was used to establish the standard curve with eight gradients of 10-fold serial dilution (10^0^–10^-7^). Three technical replicates were prepared for each sample, standard gradient, and no-template control. Amplification efficiency of all primer pairs ranged from 95% to 105%, with linear correlation coefficient (R²) values > 0.99, satisfying the requirements for absolute quantitative analysis.

### 16S *rRNA* gene amplicon sequencing analysis

Microbial DNA was extracted from ruminal fluid samples using the E.Z.N.A.® Stool DNA Kit (Omega Bio-tek, Norcross, GA, USA) according to the manufacturer’s instructions. Polymerase chain reaction amplification of bacterial *16S rRNA* genes targeting the V3–V4 hypervariable region was performed using primers 341F and 806R. For archaeal *16S rRNA* genes targeting the V4–V5 hypervariable region, amplification was conducted using primers Arch519F and Arch915R.

Sequencing libraries were generated and sequenced in PE300 mode using the MGI-G99 next-generation sequencing platform (Shanghai BIOZERON Biotech Co., Ltd., Shanghai, China) according to standard protocols. Raw sequencing reads were deposited in the NCBI Sequence Read Archive under accession number PRJNA1346834 and are publicly available.

Raw FASTQ files were initially processed for de-duplication using Trimmomatic [28], and high-quality sequences were analyzed using the DADA2 algorithm to identify insertion, deletion, and substitution errors [29]. During filtering and trimming, the expected error rate limit for each read segment was set to 2 (maxEE = 2). After sequence merging and chimera removal, representative sequence variants were classified using the RDP classifier with an 80% confidence threshold based on the Silva SSU132 database.

After standardized quality control, the average number of valid clean reads per sample was 51,761, with average read retention rates of 80.94% for the bacterial V3–V4 library and 60.99% for the archaeal V4–V5 library. A total of 6494 raw amplicon sequence variants were obtained for bacteria, with 5256 high-quality amplicon sequence variants retained after filtering. For archaea, 2233 raw amplicon sequence variants were obtained, with 1362 high-quality amplicon sequence variants retained after filtering. The rarefaction depth for diversity analysis was set to 50,000 reads to ensure complete sequencing saturation for all samples.

Rarefaction analysis was performed using Mothur software (v1.21.1) to quantify α-diversity indices including Chao1, ACE, and Shannon indices [30]. Venn diagrams were constructed using the online tool “Draw Venn Diagram” to characterize shared and unique features of amplicon sequence variants. β-diversity was assessed based on UniFrac distances, and principal coordinate analysis was conducted using the vegan package in R software.

Based on the Bray–Curtis distance matrix, the Mantel test was used to investigate correlations between microbial genera and milk production performance, fermentation characteristics, and methane-related parameters. A correlation was considered statistically significant when |Spearman’s r| > 0.6 and p < 0.05. The complete raw data matrix for Spearman correlation analysis is provided in Supplementary Table 3.

All statistical analyses were performed using the stats package in R software. One-way analysis of variance was applied to evaluate differences in diversity indices among samples, with p < 0.05 considered statistically significant. Differential abundance analysis of microorganisms was conducted using the Wilcoxon rank-sum test with Benjamini–Hochberg false discovery rate correction for multiple testing, and adjusted p < 0.05 was considered statistically significant. The complete results of microbial differential abundance analysis are presented in Supplementary Table 2.

### Statistical analysis

After preliminary organization of the data using Excel, independent sample t-tests were performed to evaluate milk production performance, apparent nutrient digestibility, serum biochemical parameters, and ruminal fermentation characteristics of dairy cows using SPSS version 27.0 (IBM Corp., Armonk, NY, USA). Data are presented as mean ± standard error of the mean, and differences were considered statistically significant at p < 0.05.

## RESULTS

### Effects of phytosterols supplementation on lactation performance and nutrient digestibility

The ingredients and nutrient composition of the diets are presented in Table 1. The effects of phytosterols supplementation on milk yield and milk composition are shown in Table 2 [22, 23]. Milk production in the PHY group was significantly higher than that in the CON group (p < 0.01). Milk fat percentage and milk protein percentage increased by 17.11% (p < 0.05) and 5.22% (p < 0.05), respectively. The addition of phytosterols had no significant effect on lactose content. Total milk solids and non-fat solids also increased by 7.10% (p < 0.05) and 3.16% (p < 0.05), respectively. ECM and 3.5% FCM in the PHY group also increased significantly (p < 0.01).

**Table 2:** Effect of feeding phytosterols on lactation performance.

Item¹	Treatment		SEM²	p-value

	CON	PHY		
DMI, kg/d	21.39	21.91	0.153	0.087
Milk yield, kg/d	25.73ᵇ	29.55ᵃ	0.640	0.002
Milk composition (%)				
Milk fat	2.98ᵇ	3.49ᵃ	0.124	0.041
Milk protein	3.45ᵇ	3.63ᵃ	0.042	0.029
Milk sugar	4.95	5.03	0.031	0.250
Total milk solids	12.25ᵇ	13.12ᵃ	0.176	0.011
Non-fat solids	9.17ᵇ	9.46ᵃ	0.060	0.014
3.5% FCM³, kg/d	23.56ᵇ	29.38ᵃ	0.863	<0.001
ECM⁴, kg/d	24.54ᵇ	30.44ᵃ	0.822	<0.001

1. DMI = Dry matter intake; FCM = Fat-corrected milk; ECM = Energy-corrected milk.

2. SEM = Standard error of least squares means.

3. 3.5% FCM = 0.432 × milk yield + 16.216 × fat yield [22].

4. ECM = 12.95 × fat yield + 7.04 × protein yield + 0.3246 × milk yield [23].

5. ᵃ^,^ᵇValues within the same row with different superscripts differ significantly (p < 0.05).

As shown in Table 3, dry matter intake did not differ significantly between the PHY and CON groups (p > 0.05). There were no significant differences in feed refusals and sorting behavior between the groups (p > 0.05). However, organic matter digestibility was 3.44% higher in the PHY group compared with the CON group (p < 0.05). In addition, the digestibility of CP, NDF, and EE increased by 2.80% (p < 0.05), 11.22% (p < 0.05), and 4.00% (p < 0.05), respectively.

**Table 3:** Effect of feeding phytosterols on nutrient digestibility and nitrogen metabolism.

Item¹	Treatment		SEM²	p-value

	CON	PHY		
Intake^3^, Kg/d				
DM	21.39	21.91	0.153	0.087
OM	20.21	20.71	0.145	0.087
CP	3.56	3.65	0.025	0.087
NDF	9.42	9.65	0.065	0.087
ADF	5.32	5.45	0.038	0.087
EE	0.56	0.53	0.017	0.087
Digestibility, %				
DM	66.98	69.07	0.643	0.105
OM	71.50^b^	73.96^a^	0.562	0.026
CP	72.08^b^	74.10^a^	0.460	0.025
NDF	48.05ᵇ	53.44ᵃ	1.077	0.010
ADF	42.20	44.12	1.050	0.369
EE	77.04ᵇ	80.12ᵃ	0.676	0.020

1. DM = Dry matter; OM = Organic matter; CP = Crude protein; NDF = Neutral detergent fiber; ADF = Acid detergent fiber; EE = Ether extract.

2. SEM = Standard error of least squares means.

3. ᵃ^,^ᵇValues within the same row with different superscripts differ significantly (p < 0.05).

### Effects of phytosterols supplementation on serum biochemical parameters

As presented in Table 4, dietary supplementation with phytosterols altered serum biochemical parameters in dairy cows. Serum glucose concentration increased by 16.92% (p < 0.05), and blood urea nitrogen increased by 38.91% (p < 0.01). Total cholesterol decreased by 20.99% (p < 0.01), and low-density lipoprotein cholesterol decreased by 31.97% (p < 0.05). Phytosterols supplementation had no significant effect on serum antioxidant indicators (p > 0.05).

**Table 4:** Effect of feeding phytosterols on serum biochemical indices and antioxidant indexes.

Item¹	Treatment		SEM²	p-value

	CON	PHY		
Biochemical index				
TP (mg/mL)	92.58	99.26	2.854	0.248
ALB (g/L)	24.64	25.34	0.438	0.433
GLU (mmol/L)	5.62ᵇ	6.57ᵃ	0.306	0.016
BUN (mmol/L)	2.75ᵇ	3.82ᵃ	0.168	0.001
TG (mmol/L)	0.15	0.17	0.009	0.193
TC (mmol/L)	5.48ᵃ	4.33ᵇ	0.179	0.001
LDL-C (mmol/L)	2.44ᵃ	1.66ᵇ	0.187	0.035
HDL-C (mmol/L)	3.41	3.26	0.215	0.729
Antioxidant index				
T-AOC (mM)	0.81	0.82	0.007	0.405
MDA (nmol/mL)	2.32	2.59	0.252	0.603
SOD (U/mL)	67.51	68.54	1.186	0.671
GSH-PX (U/mL)	110.35	109.49	5.988	0.944

1. TP = Total protein; ALB = Albumin; GLU = Glucose; BUN = Blood urea nitrogen; TG = Triglyceride; TC = Total cholesterol; LDL-C = Low-density lipoprotein cholesterol; HDL-C = High-density lipoprotein cholesterol; T-AOC = Total antioxidant capacity; MDA = Malondialdehyde; SOD = Superoxide dismutase; GSH-PX = Glutathione peroxidase.

2. SEM = Standard error of least squares means.

3. ᵃ^,^ᵇValues within the same row with different superscripts differ significantly (p < 0.05).

### Effects of phytosterols supplementation on rumen fermentation characteristics

As shown in Table 5, phytosterols supplementation significantly increased ruminal microbial CP concentration by 17.71% (p < 0.01). It had no significant effect on fermentation indicators such as pH, ammonia nitrogen, and lactic acid concentration (p > 0.05). Among the individual VFAs, acetate decreased significantly by 5.66% (p < 0.01), whereas propionate showed an increasing trend (p > 0.05). Isobutyrate, valerate, and isovalerate increased significantly by 21.92% (p < 0.01), 31.62% (p < 0.01), and 48.18% (p < 0.01), respectively. The acetate-to-propionate ratio decreased significantly by 13.25% (p < 0.05), whereas no significant changes were observed in butyrate or total VFA concentrations. Phytosterols supplementation increased ruminal bacterial copy number by 2.81% (p < 0.05), whereas archaeal copy number concentration decreased by 2.27% (p < 0.01).

**Table 5:** Effect of feeding phytosterols on rumen fermentation parameters and microbial populations.

Item¹	Treatment		SEM²	p-value

	CON	PHY		
pH	6.46	6.38	0.044	0.347
NH₃-N (mg/mL)	8.39	8.51	0.902	0.92
Total VFA (mmol/L)	111.51	115.91	3.188	0.499
Acetate (mmol/L)	67.01	65.88	1.965	0.778
Propionate (mmol/L)	27.52	30.96	0.935	0.065
Butyrate (mmol/L)	13.47	14.04	0.711	0.697
Isobutyrate (mmol/L)	0.81ᵇ	1.03ᵃ	0.041	0.004
Valerate (mmol/L)	1.50ᵇ	2.01ᵃ	0.08	0.001
Isovalerate (mmol/L)	1.21ᵇ	1.93ᵃ	0.138	0.007
VFA, % molar proportion				
Acetate	60.12ᵃ	56.72ᵇ	0.525	0.001
Propionate	24.7	27.07	0.666	0.075
Butyrate	11.98	11.9	0.42	0.922
Isobutyrate	0.73ᵇ	0.89ᵃ	0.029	0.004
Valerate	1.36ᵇ	1.79ᵃ	0.054	0.001
Isovalerate	1.10ᵇ	1.63ᵃ	0.084	0.001
Acetate:Propionate	2.49ᵃ	2.16ᵇ	0.078	0.034
Lactic acid (mmol/L)	1.88	1.76	0.109	0.603
MCP (mg/dL)	154.34ᵇ	181.68ᵃ	4.422	0.001
Bacteria Total (log10⁸/mL)	11.37ᵇ	11.69ᵃ	0.068	0.013
Archaea Total (log10⁶/mL)	7.48ᵃ	7.31ᵇ	0.034	0.008

1. NH₃-N = Ammonia nitrogen; VFA = Volatile fatty acid; MCP = Microbial crude protein.

2. SEM = Standard error of least squares means.

3. ᵃ^,^ᵇValues within the same row with different superscripts differ significantly (p < 0.05).

### Effects of phytosterols supplementation on enteric gas emissions

This part of the experiment was conducted using seven cows per group. Compared with the CON group, CH_4_ production per kilogram of ECM in the PHY group decreased by 14.42% (p < 0.05). Similarly, CH_4_ (p > 0.05) and CO_2_ (p > 0.05) emissions per kilogram of milk yield also showed a decreasing trend (Table 6).

**Table 6:** Effect of feeding phytosterols on methane emission parameters.

Item¹	Treatment		SEM²	p-value

	CON	PHY		
CH₄, g/d	424.34	415.43	14.303	0.769
CO_2_, g/d	16,057.84	15,893.55	495.237	0.876
Methane-equivalent emissions, g/kg				
CH_4_/DMI	19.71	19.35	0.690	0.808
CH_4_/MY	14.97	13.19	0.492	0.068
CH_4_/ECM	15.39^a^	13.17^b^	0.573	0.048
Carbon dioxide-equivalent emissions, g/kg				
CO_2_/DMI	747.42	740.97	26.63	0.909
CO_2_/MY	566.03	504.93	16.486	0.060
CO_2_/ECM	583.21	507.26	23.693	0.111

1. CH₄ = Methane; DMI = Dry matter intake; CO₂ = Carbon dioxide; H₂ = Hydrogen; GE = Gross energy.

2. SEM = Standard error of least squares means.

3. ᵃ^,^ᵇValues within the same row with different superscripts differ significantly (p < 0.05).

### Effects of phytosterols supplementation on ruminal microbial diversity

**Figure 1:**
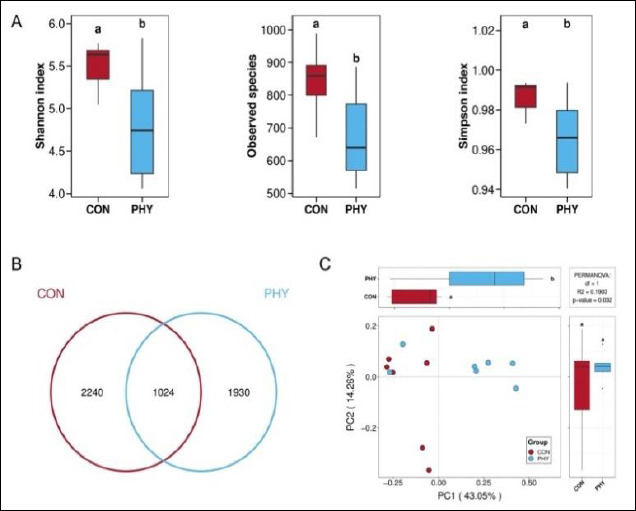
Effects of phytosterols supplementation on ruminal microbial diversity and community structure in dairy cows. (A) Comparison of α-diversity indices between the CON and PHY groups, including Shannon index, observed species richness, and Simpson index. Different lowercase letters indicate significant differences within each index (p < 0.05). (B) Venn diagram showing the shared and unique amplicon sequence variants between the CON and PHY groups. (C) Principal coordinate analysis based on Bray–Curtis dissimilarity illustrating β-diversity differences between microbial communities of the CON and PHY groups. Boxplots on the right represent the distribution of samples along PC1 and PC2.

As shown in Figure 1A, α-diversity indices were lower in the PHY group than in the CON group. The PHY group exhibited reduced Shannon diversity, observed species richness, and Simpson index values. The Venn diagram illustrated that the CON and PHY groups shared 1,024 amplicon sequence variants, whereas 2,240 amplicon sequence variants were unique to the CON group and 1,930 amplicon sequence variants were unique to the PHY group (Figure 1B). β-diversity analysis based on principal coordinate analysis (Figure 1C) revealed clear separation between the CON and PHY groups along PC1, which accounted for 43.05% of the total variance. Permutational multivariate analysis of variance confirmed that the overall ruminal microbial community structure differed significantly between treatments (R² = 0.1903; p = 0.032).

### Effects of phytosterols supplementation on ruminal bacterial composition

**Figure 2:**
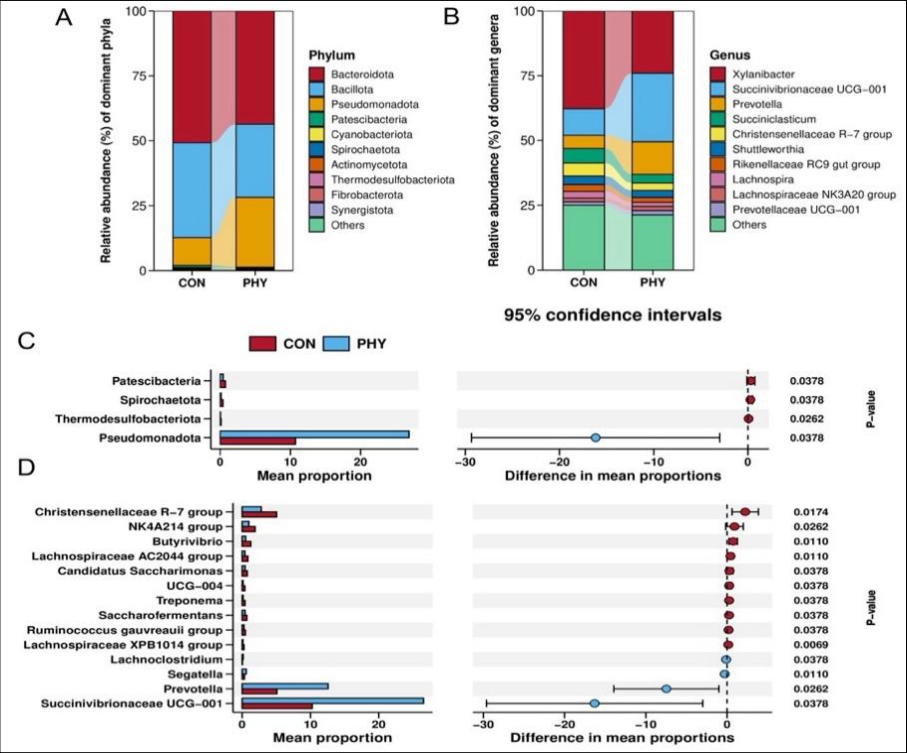
Effects of phytosterols supplementation on ruminal bacterial composition and differential abundance in dairy cows. (A–B) Relative abundance of dominant bacterial phyla and genera in the CON and PHY groups. (C–D) Differential abundance analyses at the phylum and genus levels showing mean proportions (left) and differences in mean proportions with 95% confidence intervals (right) between the two dietary treatments. Points located to the left or right of the zero line indicate lower or higher abundance, respectively, in the PHY group compared with the CON group.

Figure 2A illustrates the dominant bacterial phyla in the CON and PHY groups, with Bacteroidota, Bacillota, and Pseudomonadota representing the top three phyla. As shown in Figure 2C, the relative abundance of Pseudomonadota was significantly higher in the PHY group, whereas Patescibacteria, Spirochaetota, and Thermodesulfobacteriota exhibited significantly lower relative abundances compared with the CON group.

Figure 2B presents the dominant genera in both groups, with *Xylanibacter*, *Succinivibrionaceae* UCG-001, and *Prevotella* identified as the top three genera. The relative abundances of *Succinivibrionaceae* UCG-001, *Prevotella*, *Segatella*, and *Lachnoclostridium* were significantly higher in the PHY group compared with the CON group. In contrast, the relative abundances of *Christensenellaceae* R-7 group, NK4A214 group, *Butyrivibrio*, *Lachnospiraceae* AC2044 group, *Candidatus Saccharimonas*, UCG-004, *Treponema*, *Saccharofermentans*, *Ruminococcus gauvreauii* group, and *Lachnospiraceae* XPB1014 group were significantly lower in the PHY group compared with the CON group.

### Effects of phytosterols supplementation on ruminal archaeal composition

**Figure 3:**
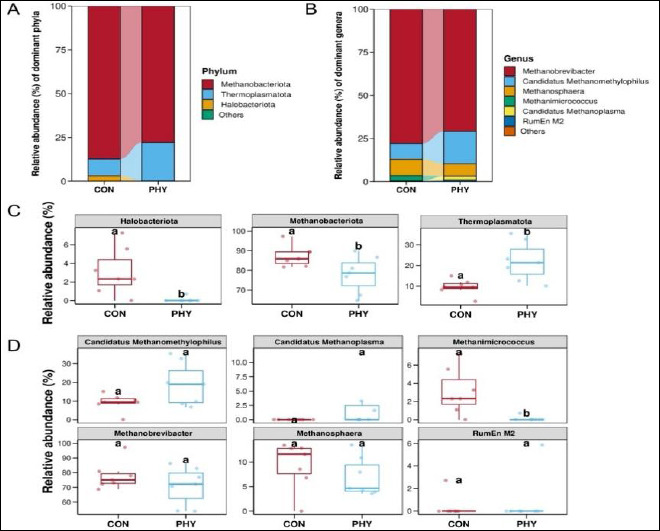
Effects of phytosterols supplementation on ruminal archaeal composition and differential abundance in dairy cows. (A–B) Relative abundance of dominant archaeal phyla and genera in the CON and PHY groups. (C–D) Boxplots showing between-group differences in archaeal relative abundances at the phylum and genus levels. Statistical comparisons were performed using t-tests. Different lowercase letters above the boxes indicate significant differences between the CON and PHY groups (p < 0.05).

As illustrated in Figure 3A, the dominant archaeal phyla in the rumen were primarily Methanobacteriota and Thermoplasmatota. At the genus level, the major representatives included *Methanobrevibacter*, *Candidatus Methanomethylophilus*, and *Methanosphaera* (Figure 3B). As shown in Figure 3C, phytosterols supplementation significantly increased the relative abundance of Thermoplasmatota while decreasing the abundances of Halobacteriota and Methanobacteriota. At the genus level, phytosterols supplementation reduced the relative abundance of *Methanimicrococcus* (Figure 3D).

### Correlation analysis between ruminal microorganisms and production traits

**Figure 4:**
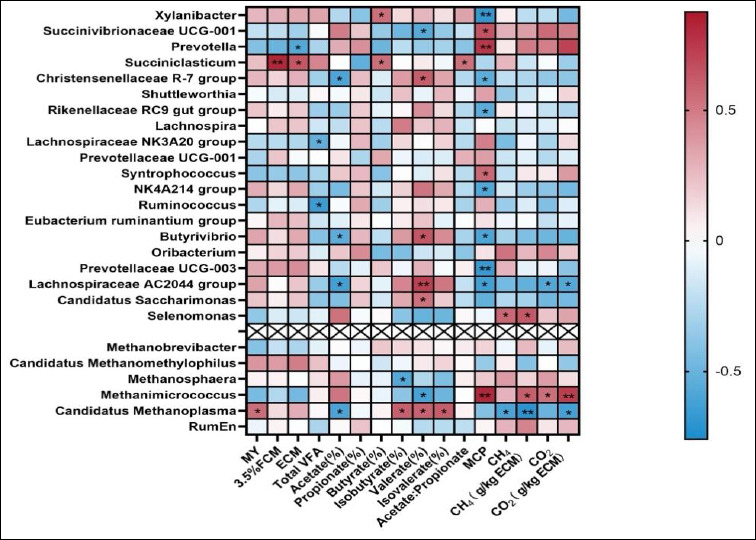
Spearman correlation analysis of ruminal bacterial and archaeal genera with dairy production performance, ruminal fermentation characteristics, and methane-related parameters.

Correlation heatmaps were constructed using abundance data of the top 20 bacterial genera and archaeal genera in relation to dairy production performance, ruminal fermentation characteristics, and methane-related parameters, as presented in Figure 4.

In the heatmap, positive and negative correlations are represented by blue and red gradients, respectively, with color intensity increasing according to correlation strength. Statistically significant correlations are indicated by asterisks (*p < 0.05; **p < 0.01). Taxa above the crosshatched diagonal cells represent bacterial genera, whereas taxa below the diagonal represent archaeal genera.

## DISCUSSION

### Effects of phytosterols supplementation on lactation performance and serum biochemical parameters

According to Lv *et al*. [16], dietary supplementation with phytosterols increased the energy utilization efficiency of perinatal dairy cows. Moreover, supplementation with 200 mg/d phytosterols in perinatal cows increased milk yield by 1.82 kg/d [14], which is consistent with the increase in milk production observed in the present study. Notably, the effective phytosterol dose used in the present study (15 g/d commercial product = 750 mg/d active phytosterols) was higher than the 200 mg/d dose commonly used in previously published dairy cow trials, which mainly focused on perinatal cows. This higher dose may be required in mid-lactation cows with higher dry matter intake and a more stable metabolic state to induce measurable alterations in ruminal fermentation and methanogenesis; however, this interpretation requires further dose-response validation.

The results of the present study demonstrated that the concurrent increases in milk yield and apparent nutrient digestibility suggest that phytosterols may optimize the partitioning of dietary energy toward lactation rather than adipose tissue deposition. Gao *et al*. [14] reported that phytosterols increased plasma concentrations of free fatty acids such as C16:0 and C18:0 in dairy cows. These fatty acids also participate in milk fat synthesis within the mammary gland [31]. These findings are consistent with the elevated milk fat percentage observed in the PHY group in the present study.

Interestingly, the acetate proportion in the PHY group showed a marked reduction (p = 0.001), which appears paradoxical considering the concurrent increase in milk fat content. However, this apparent discrepancy may be explained by alterations in ruminal fermentation dynamics and microbial–host metabolic interactions. A lower acetate proportion does not necessarily indicate a decrease in its absolute concentration because the absolute ruminal acetate concentration did not differ significantly between groups (p = 0.778). Additional support is provided by previous *in vitro* studies indicating that phytosterols supplementation stimulates ruminal acetate production [15].

These observations suggest that phytosterols may simultaneously enhance acetate synthesis and promote acetate absorption and utilization by the host animal. Under *in vivo* conditions, phytosterols may improve the absorptive capacity of the ruminal epithelium, thereby facilitating more efficient transfer of acetate into the bloodstream. Consequently, enhanced activation of lipogenic pathways in mammary tissue may increase utilization of circulating acetate as a substrate for milk fat synthesis. This dual mechanism involving increased acetate absorption and enhanced mammary utilization may explain the inverse relationship observed between ruminal acetate proportion and milk fat content in the present study. Nevertheless, this remains a working hypothesis based on observational findings, and future mechanistic studies are required to directly verify the regulatory effects of phytosterols on ruminal epithelial function.

Available evidence suggests that alteration of the phytosterol profile of bovine milk through dietary supplementation has only a limited effect, with total phytosterol concentrations in milk remaining extremely low (<0.12 mg/100 mL) even under high-phytosterol feeding conditions [32]. Therefore, substantial phytosterol enrichment of milk is unlikely under practical feeding conditions. However, because milk sterol profiles were not quantified in the present study, future investigations should determine whether phytosterols or their metabolites are transferred into milk and whether such alterations may provide nutritional, marketing, or consumer perception advantages [32].

Biochemical analysis demonstrated that phytosterols supplementation increased the availability of gluconeogenic substrates and improved lipid metabolism. Phytosterols may indirectly influence glucose metabolism by enhancing the availability of gluconeogenic precursors such as propionate [14, 16]. The significantly increased abundance of *Prevotella* and *Segatella* in the PHY group may enhance carbohydrate fermentation and propionate production, thereby increasing blood glucose concentrations through the gluconeogenic pathway [33]. Meanwhile, enrichment of Pseudomonadota, including *Succinivibrionaceae* UCG-001, may promote succinate metabolism and indirectly enhance glucose synthesis [34].

The elevated blood urea nitrogen concentration observed in the PHY group suggests enhanced nitrogen flux resulting from increased ruminal microbial proteolysis and subsequent ammonia absorption [35]. The increase in milk protein percentage and improved protein digestibility further support this interpretation. In combination with the observed increases in NDF and organic matter digestibility, these findings suggest that phytosterols improved overall nitrogen recycling efficiency and nitrogen utilization, thereby supporting milk protein synthesis.

Regarding lipid metabolism, the significant reductions in serum total cholesterol and low-density lipoprotein cholesterol are consistent with the established cholesterol-lowering properties of phytosterols. Mechanistically, phytosterols compete with cholesterol for incorporation into mixed micelles within the intestinal lumen, thereby reducing cholesterol absorption and potentially increasing hepatic low-density lipoprotein receptor expression to enhance cholesterol clearance [13, 36].

In contrast to previous studies that reported significant antioxidant effects of phytosterols, including increased superoxide dismutase and glutathione peroxidase activities together with decreased malondialdehyde concentrations [37, 38], the present study did not observe significant alterations in antioxidant indices. This discrepancy may be associated with differences in the physiological status of the experimental animals. Most studies reporting antioxidant benefits involved animals exposed to specific stress conditions such as heat stress, lipopolysaccharide challenge, or high-grain feeding, where basal oxidative stress levels are elevated.

In the present study, the cows were maintained under relatively stable physiological conditions without severe oxidative stress challenges. Therefore, endogenous antioxidant enzyme activities may already have been maintained at normal physiological levels, reducing the requirement for additional antioxidant support from phytosterols [39]. Furthermore, the dose-response relationship of phytosterols appears to be complex. Although 200 mg/d phytosterols has previously been shown to be effective [14], higher supplementation levels such as the 750 mg/d active phytosterols used in the present study may trigger different metabolic feedback mechanisms. Consequently, the absence of significant changes in antioxidant enzyme activities in the present study likely reflects physiological redox homeostasis rather than lack of phytosterol bioactivity.

### Effects of phytosterols supplementation on rumen fermentation characteristics

Dietary supplementation with phytosterols can alter ruminal fermentation patterns and modulate the ruminal microbial community [14]. Xi *et al*. [15] reported that phytosterols supplementation during *in vitro* fermentation increased total VFA and microbial CP concentrations. Similarly, Lv *et al*. [16] demonstrated that supplementation with 200 mg/d phytosterols increased total bacterial copy number and microbial CP concentration in the rumen. The findings obtained in the present study are generally consistent with these previous reports.

Phytosterols may enhance ammonia nitrogen utilization efficiency by stimulating the growth of proteolytic bacteria such as *Segatella* [40]. In the present study, acetate proportion and acetate-to-propionate ratio decreased significantly. Although the increase in propionate proportion was not statistically significant (p = 0.075), the trend suggests a shift away from acetate production toward propionate formation. This shift is metabolically advantageous because propionate functions as a major hydrogen sink within the rumen, competing with methanogens for hydrogen and thereby reducing the hydrogen available for methanogenesis, which likely contributed to the reduction in methane emission intensity observed in this study [41].

This hydrogen competition mechanism is further supported by previous reviews indicating that propionate synthesis competes directly with methanogenesis for metabolic hydrogen in the rumen, and that redirecting hydrogen toward propionate production may represent an effective strategy for methane mitigation while maintaining productivity [42, 43]. The observed shift in fermentation pattern was closely associated with the increased abundance of *Succinivibrionaceae* UCG-001. Phytosterols may promote propionate synthesis by enriching propionate-producing bacteria such as *Succinivibrionaceae* UCG-001. In addition, *Prevotella*, which functions as a major sugar-degrading bacterium, may also enhance propionate production [17].

Furthermore, the relative abundance of Pseudomonadota increased significantly in the PHY group. This phylum contains several taxa involved in propionate metabolism, such as *Succinivibrio*, and its enrichment was associated with the increased propionate trend (p = 0.075) and decreased acetate-to-propionate ratio [44]. Moreover, the decreased abundance of *Butyrivibrio* may further reduce acetate production and thereby decrease acetate proportion [17]. Similar shifts in CH_4_-linked fermentation characteristics, including reduced acetate proportion, have been reported in studies evaluating essential oils, seaweed, and 3-nitrooxypropanol supplementation [45, 46], supporting the functional relevance of the present findings.

For comparison, a meta-analysis evaluating a commercial essential-oil blend in lactating cows reported an average 10% reduction in methane production intensity without affecting dry matter intake, whereas 3-nitrooxypropanol generally achieves >30% reductions in CH_4_ yield and intensity. However, very high doses of 3-nitrooxypropanol may negatively affect dry matter intake and ECM production in some studies [47–49].

Isobutyric acid and isovaleric acid are branched-chain VFAs derived from amino acid deamination and generally indicate accelerated microbial fermentation of protein substrates [50]. Increased isobutyric acid concentration may reflect enhanced protein fermentation and microbial turnover associated with peptide and amino acid catabolism [47]. The significant increase in branched-chain VFA concentrations observed in the present study may indirectly enhance NDF digestibility by functioning as growth factors for fibrolytic bacteria.

The increased abundance of *Prevotella* may also promote valeric acid synthesis through glycolytic pathways [17]. In addition, *Prevotella* can contribute to the synthesis of branched-chain fatty acids such as isovaleric acid and isobutyric acid through amino acid deamination pathways. The reduced abundance of *Butyrivibrio* and *Lachnospiraceae* may further contribute to enhanced branched-chain VFA synthesis [42].

### Regulatory effects of phytosterols supplementation on ruminal bacterial and archaeal communities

One of the most important findings of the present study was the reduction in methane emission intensity (CH_4_/ECM), which was associated with substantial alterations in ruminal fermentation characteristics and microbial community structure. Because absolute CH_4_ output (g/d) did not differ significantly between treatments, the primary response should be interpreted as improved production efficiency through reduced CH_4_ intensity rather than direct suppression of total methane production.

Phytosterols may reduce methane production through dual mechanisms. First, the reduction in acetate proportion may decrease the abundance of hydrogen-utilizing methanogens, whereas competitive hydrogen utilization by propionate-producing bacteria such as *Succinivibrionaceae* UCG-001 may further reduce methanogenic substrate availability. Second, methane production may be indirectly inhibited through reduction of bacterial taxa that provide enzymes and electrons required for methanogenesis, including *Christensenellaceae* R-7 group.

Lv *et al*. [16] reported that supplementation with 200 mg/d phytosterols exerted no significant effect on α-diversity of ruminal bacteria, which differs from the findings of the present study. The reduced α-diversity observed here may therefore represent a dose-dependent or physiological-stage-dependent response associated with mid-lactation cows. Importantly, reduced α-diversity does not necessarily indicate impaired microbial function. For example, monensin supplementation has been shown to reduce ruminal α-diversity while simultaneously reshaping the microbial ecosystem toward greater carbohydrate degradation efficiency [51].

β-diversity analysis using principal coordinate analysis revealed clear separation between the PHY and CON groups, indicating that phytosterols supplementation substantially remodeled the ruminal microbial community. These changes were directly associated with shifts in the abundance of core microbial taxa [52].

At the bacterial community level, although *Bacillota* and *Bacteroidota* remained the dominant phyla, several notable alterations were observed. Increased abundance of *Succinivibrionaceae* UCG-001, *Prevotella*, and *Lachnoclostridium* in the PHY group may promote sugar fermentation and short-chain fatty acid production [53]. *Succinivibrionaceae* UCG-001 converts phosphoenolpyruvate to propionate through the oxaloacetate pathway, thereby enhancing hydrogen utilization and reducing the availability of methanogenic substrates [54].

Meanwhile, enhanced metabolism of branched-chain VFAs further consumes reducing equivalents such as H_2_ [55]. This shift in metabolic hydrogen utilization may reduce hydrogen accumulation available for methane production, thereby contributing directly to reduced methane emission intensity [56]. Hydrogen-producing bacterial taxa such as *Christensenellaceae* R-7 group and *Butyrivibrio* decreased in abundance. Previous studies have demonstrated that *Christensenellaceae* R-7 group can directly transfer electrons to methanogens and that [FeFe]-hydrogenases produced by this group may promote hydrogenotrophic methane synthesis [57]. Zhao *et al*. [58] also reported a significant association between *Christensenellaceae* R-7 group abundance and methane production, further supporting the methane-reducing effect observed in the present study [54].

Methane production in the rumen is primarily driven by hydrogenotrophic archaea belonging to the phylum *Methanobacteriota*, formerly classified as Euryarchaeota, with *Methanobrevibacter* accounting for up to 74% of the archaeal population [59]. Therefore, the observed reduction in *Methanobacteriota* abundance and the decreased archaeal copy number may directly contribute to reduced methane production intensity.

The present study provides novel evidence that phytosterols can serve as an effective feed additive for mid-lactation dairy cows by simultaneously enhancing productive performance and reducing enteric methane intensity through modulation of ruminal microbial pathways. Unlike previous studies limited to perinatal cows or *in vitro* systems, the present study demonstrated that supplementation with a commercial high-dose phytosterols product improved ECM yield and milk solids while reducing CH_4_/ECM without negatively affecting dry matter intake, representing a favorable combination rarely achieved among methane mitigation strategies.

Compared with essential oils or 3-nitrooxypropanol, which may achieve larger reductions in methane emissions but occasionally depress feed intake or milk production, phytosterols supplementation achieved moderate methane mitigation together with positive lactation responses [48, 49]. The tested supplementation level (750 mg active phytosterols/d) was substantially higher than doses used in previous ruminant studies (200 mg/d), suggesting that stronger microbial modulation and methane-related responses may require higher supplementation levels than previously evaluated.

### Study limitations and future directions

Several limitations of the present study should be acknowledged. First, the relatively short experimental duration precluded evaluation of long-term effects on reproductive performance, animal health, and sustained methane mitigation responses. Second, the relatively small subsample size used for methane measurements and microbial sequencing may have limited statistical power and reduced the generalizability of the findings.

Furthermore, only a single phytosterols product and supplementation level were evaluated, preventing establishment of a definitive dose-response relationship. The bioavailability of supplemented phytosterols was also not determined, and the absence of a separate vehicle control group made it difficult to completely exclude potential confounding effects associated with the carrier material.

Future studies should therefore include larger sample sizes, longer experimental durations, and optimized experimental designs to further clarify the mechanisms underlying phytosterols-mediated methane mitigation. Integration of multi-omics approaches may also improve understanding of the molecular interactions between phytosterols, ruminal microorganisms, and host metabolism. In addition, future investigations should include economic evaluations under commercial dairy farming conditions to determine the practical feasibility of phytosterols supplementation strategies.

## CONCLUSION

Dietary supplementation with a β-sitosterol-rich phytosterols product at 15 g/d positively influenced lactation performance, nutrient digestibility, ruminal fermentation characteristics, and ruminal microbial composition in mid-lactation Holstein dairy cows. Phytosterols supplementation significantly increased milk yield, milk fat percentage, milk protein percentage, ECM, and FCM without affecting dry matter intake. Improvements in apparent digestibility of organic matter, CP, NDF, and EE further indicated enhanced nutrient utilization efficiency. In addition, serum glucose concentration increased, whereas total cholesterol and low-density lipoprotein cholesterol decreased, suggesting improved energy metabolism and lipid regulation.

Phytosterols supplementation also altered ruminal fermentation patterns by decreasing acetate proportion and acetate-to-propionate ratio while increasing microbial CP concentration and branched-chain VFA production. These fermentation shifts were accompanied by substantial remodeling of the ruminal bacterial and archaeal communities. Increased abundances of *Succinivibrionaceae* UCG-001, *Prevotella*, and other propionate-associated bacterial taxa, together with decreased abundance of methanogenic archaea, particularly *Methanobacteriota*, suggest that phytosterols redirected metabolic hydrogen away from methanogenesis toward more energetically efficient fermentation pathways. Consequently, CH_4_ emission intensity expressed as CH_4_/ECM decreased significantly in the PHY group.

From a practical perspective, the present findings suggest that phytosterols may represent a promising nutritional strategy for improving dairy production efficiency while simultaneously reducing the environmental impact of enteric methane emissions. Unlike several methane mitigation additives that may negatively affect feed intake or milk production, phytosterols supplementation in this study improved productive performance without depressing dry matter intake, highlighting its potential applicability under commercial dairy production systems.

One of the major strengths of this study was the integrated experimental approach combining production performance evaluation, nutrient digestibility assessment, serum biochemical analysis, methane emission measurement, ruminal fermentation characterization, qPCR quantification, and *16S rRNA* gene sequencing analysis. This comprehensive evaluation provided detailed insight into the interactions among phytosterols supplementation, ruminal microbial ecology, fermentation pathways, and methane emission intensity in lactating dairy cows.

Nevertheless, several limitations should be considered. The relatively short experimental period limited evaluation of long-term physiological responses and production sustainability. In addition, the relatively small sample size for methane measurements and microbial sequencing may have reduced statistical power. Furthermore, only a single phytosterols dose and product formulation were evaluated, preventing establishment of a definitive dose-response relationship.

Future studies should therefore investigate the long-term effects of phytosterols supplementation under commercial dairy production conditions, evaluate multiple supplementation levels, and integrate multi-omics approaches to further elucidate the molecular and microbial mechanisms involved in methane mitigation. Additional studies examining economic feasibility, ruminal epithelial metabolism, and potential transfer of phytosterols into milk would also strengthen the practical application value of phytosterols supplementation strategies.

In conclusion, supplementation with high-dose phytosterols improved lactation performance, enhanced nutrient utilization, modulated ruminal microbial communities, and reduced methane emission intensity in mid-lactation dairy cows. These findings provide important evidence supporting phytosterols as a potentially sustainable feed additive for simultaneously improving dairy productivity and mitigating environmental impacts associated with enteric methane emissions.

## DATA AVAILABILITY

The *16S rRNA* gene sequencing data generated in this study have been deposited in the NCBI Sequence Read Archive under accession number PRJNA1346834 and are publicly available. All other raw data supporting the findings of this study are available from the corresponding author upon reasonable request.

## AUTHOR’S CONTRIBUTIONS

DL, MW and YC: Study conception and design. YK, QW, JG, RW, MW, CD and DLv: Performed material preparation, data collection and analysis, and revised the manuscript. DL: Drafted the manuscript. MW, WZ and YC: Supervised and designed the study and reviewed the manuscript. All authors have read and approved the final version of the manuscript.
